# Artificial intelligence-enabled early warning systems for public health preparedness: perspectives of senior public health leaders in a Small Island Developing State

**DOI:** 10.3389/fpubh.2026.1931303

**Published:** 2026-08-13

**Authors:** Letetia Addison, Shalini Pooransingh, Loren De Freitas

**Affiliations:** The University of the West Indies St. Augustine, St. Augustine, Trinidad and Tobago

**Keywords:** artificial intelligence, climate and health education, early warning systems, health system preparedness and response, leadership perspectives, public health education and health promotion, Small Island Developing States

## Abstract

**Purpose:**

Artificial intelligence-enabled early warning systems (AI-EWS) are increasingly recognised as tools for strengthening public health preparedness and education in climate-vulnerable settings. However, limited empirical evidence exists on how public health leaders perceive their use in practice. This study aimed to examine the perspectives of senior public health leaders, specifically County Medical Officers of Health (CMOHs), on AI-EWS in Trinidad and Tobago.

**Materials and methods:**

An exploratory descriptive study was conducted using a structured survey of County Medical Officers of Health, with six of nine CMOHs completing the questionnaire (response rate = 66.7%). The survey assessed familiarity, perceived usefulness, system priorities, institutional readiness, and implementation barriers. Data were analysed descriptively using frequencies and proportions, with open-ended responses summarised using inductive thematic categorisation.

**Results:**

All respondents (6/6) identified infectious diseases and flooding as priority applications for AI-EWS, while three (3/6) identified heat-related risks. Key system features, including dashboards (5/6), integration with emergency services (5/6), and automated alerts (3/6), were widely perceived as useful. Equity was prioritised by all respondents (6/6), particularly for underserved populations. However, barriers were also reported, including budget constraints (5/6), limited technical capacity (3/6), and data challenges (3/6).

**Conclusion:**

AI-EWS are perceived as valuable tools for supporting public health decision-making, coordination, and professional learning in SIDS contexts. However, successful implementation will require strengthening infrastructure, workforce capacity, governance frameworks, and equitable system design. These findings provide early empirical insight to inform the responsible integration of AI-enabled systems into public health education and preparedness.

## 1 Introduction

Small Island Developing States (SIDS) are increasingly exposed to climate-sensitive hazards, including flooding, extreme rainfall, heatwaves, tropical cyclones, and sea level rise, with significant implications for population health and health system resilience (1, 2). These hazards contribute to increased risks of vector-borne and waterborne diseases, disruptions to health service delivery, and additional strain on already resource-constrained public health systems in Caribbean contexts (2). Regional evidence has further highlighted the climate-health nexus in the Caribbean, including risks related to dengue, heat exposure, waterborne disease, and the need for stronger climate-health surveillance and response capacity (3).

The structural characteristics of SIDS, including small population size, geographic exposure, economic vulnerability, and limited technical capacity, further compound their susceptibility to climate-related health risks (4). In Trinidad and Tobago, climate-related health concerns include flooding, heat-related risks, vector-borne diseases, and the need to strengthen health system preparedness for climate-sensitive hazards (5). Strengthening public health preparedness in these settings therefore requires not only robust surveillance systems, but also enhanced leadership capacity, institutional learning, and effective risk communication mechanisms. Understanding institutional readiness for AI-enabled systems is therefore critical for informing their effective integration into public health education and decision-making.

Early warning systems (EWS) are widely recognised as cost-effective tools for reducing disaster-related health impacts by providing timely and actionable information that enables anticipatory decision-making and response (6). This is consistent with the Sendai Framework for Disaster Risk Reduction 2015–2030, which emphasises strengthening disaster risk governance, preparedness, and access to multi-hazard early warning systems (7). In public health emergencies, systems such as the World Health Organization’s Early Warning, Alert and Response System support the rapid detection of outbreaks and facilitate timely interventions to limit disease transmission and reduce mortality (8). Global initiatives such as the United Nations “Early warnings for all” programme further emphasise the importance of integrated, multi-hazard, people-centred systems that connect risk knowledge, forecasting, communication, and response preparedness (6).

For the purposes of this study, AI-enabled early warning systems are understood as data-driven systems that use artificial intelligence methods, including machine learning, predictive analytics, natural language processing, or other automated pattern-recognition approaches, to enhance the detection, forecasting, communication, and response coordination functions of traditional early warning systems (9–12). In public health, AI-EWS may combine epidemiological, clinical, environmental, meteorological, mobility, laboratory, or community-based data to identify unusual patterns, anticipate emerging risks, generate alerts, and support decision-makers in prioritising timely action (9, 10). Applications discussed in the literature include infectious disease surveillance, outbreak detection, syndromic monitoring, climate-sensitive disease forecasting, risk mapping, automated alerts, decision support, and emergency preparedness (9–12).

During and after the COVID-19 pandemic, interest in AI-enabled public health surveillance and decision-support tools increased as health systems sought more adaptive ways to integrate diverse data streams, monitor rapidly changing risks, and support decision-making under uncertainty (10–13). However, evidence on the design and implementation of AI-EWS in Caribbean SIDS remains limited, particularly from the perspective of senior public health decision-makers responsible for surveillance, response coordination, and community-level preparedness.

Building on these developments, recent advances in AI have expanded the practical capabilities of early warning systems in public health. AI-enabled systems can support real-time data processing, pattern recognition, outbreak detection, risk prediction, and decision support, particularly in the context of infectious disease surveillance and climate-related risks (9–12). In addition to strengthening forecasting capacity, AI has been identified as a tool for improving resource allocation, risk communication, coordination, and public health education (12, 14).

Despite these advances, significant challenges remain regarding the implementation of AI-enabled systems in public health. These include issues related to data quality, infrastructure limitations, governance and regulatory frameworks, workforce capacity, and ethical considerations such as privacy, transparency, and equity (10, 12). Health equity considerations are particularly important because AI systems may reproduce or amplify existing disparities if data, design, implementation, and governance processes do not explicitly account for underserved populations and structural inequities (15). These challenges are particularly pronounced in SIDS contexts, where digital infrastructure and technical expertise may constrain the adoption and effective use of advanced technologies. At the same time, the potential role of AI-enabled early warning systems as tools for public health education, leadership decision-making, and institutional preparedness remains underexplored.

Empirical evidence on the perspectives of frontline public health decision-makers in SIDS is limited. In particular, there is a lack of research examining how senior public health officials, who play a central role in surveillance, response coordination, and public health decision-making within the Trinidad and Tobago health system, perceive the value, challenges, and implementation pathways for AI-enabled early warning systems. This gap is important because emerging evidence suggests that AI may support public health decision-making in both routine and crisis contexts, but its practical use by public health leaders and policy actors remains underdeveloped (13). Understanding these perspectives is critical for informing context-appropriate system design, capacity-building strategies, and responsible implementation.

This brief research report addresses this gap by examining senior public health officials’ perspectives on AI-enabled early warning systems in Trinidad and Tobago. Specifically, the study explores perceived applications, system priorities, ethical and governance considerations, and institutional readiness for adoption. By providing early empirical insight from a specialised leadership population, the study contributes to emerging discussions on the role of AI-enabled systems in strengthening public health education, preparedness, and resilience in climate-vulnerable SIDS contexts.

## 2 Materials and methods

### 2.1 Study setting

The study was conducted in Trinidad and Tobago, a twin-island, Small Island Developing State in the Caribbean. The national public health system is governed by the Ministry of Health, with healthcare delivery managed through Regional Health Authorities (RHAs). County Medical Officers of Health (CMOHs) serve as senior public health leaders within this system and are responsible for disease surveillance, outbreak response coordination, health promotion, and community-level public health decision-making. In Trinidad and Tobago, there are a total of 9 CMOHs across both islands.

The country is regularly exposed to climate-sensitive hazards, including flooding, extreme rainfall, and heat events, which increase the risk of infectious diseases and place pressure on public health systems. This setting provides a relevant context for examining leadership perspectives on the adoption and use of artificial intelligence-enabled early warning systems (AI-EWS) in public health.

### 2.2 Study design

This study employed an exploratory descriptive design using a structured survey to examine perspectives on artificial intelligence-enabled early warning systems (AI-EWS) among County Medical Officers of Health (CMOHs) in Trinidad and Tobago.

The study aimed to generate early empirical insight from a small and specialised leadership population, focusing on perceived system usefulness, institutional readiness, training needs, and implementation challenges. The survey included primarily closed-ended items, with a limited number of open-ended questions included to provide additional contextual insight. Given the exploratory nature of the study and the small sample size, the design was intended to support descriptive analysis rather than inferential generalisation.

### 2.3 Target population and sampling

The target population comprised all nine County Medical Officers of Health (CMOHs) in Trinidad and Tobago. These individuals represent the most senior public health physicians operating at the regional level within the Ministry of Health system. A purposive sampling approach was used, with all nine CMOHs invited to participate in the study. This approach reflects a total population inclusion strategy within a highly specialised leadership group rather than a representative sample of a broader population. Six CMOHs completed the survey, resulting in a response rate of 66.7%.

### 2.4 Data collection

Data were collected using a structured online questionnaire administered between February and April 2026. The survey instrument was developed *de novo* to capture leadership perspectives on the use of artificial intelligence-enabled early warning systems in public health. The questionnaire design was informed by existing literature and conceptual frameworks related to artificial intelligence readiness, public health preparedness, and surveillance systems. In particular, the instrument was aligned with key domains commonly identified in AI readiness and public health system assessment tools, including governance, infrastructure, data systems, workforce capacity, ethics, and implementation readiness (16).

Items were designed to reflect these domains while incorporating context-specific considerations relevant to SIDS settings and leadership decision-making. The instrument comprised 20 core items, including 15 closed-ended and 5 open-ended items organised into thematic domains, including:

- Leadership experience and current monitoring practices.
- Familiarity with AI-enabled early warning systems.
- Priority public health threats.
- Perceived usefulness of system features.
- Design priorities and system characteristics.
- Ethical, governance, and equity considerations.
- Institutional readiness and training needs.
- Partnerships and implementation pathways.
- Perceived barriers and enablers.

Closed-ended items included Likert-scale questions, ranking exercises, and multiple-response items. Open-ended questions allowed respondents to elaborate on system design considerations, capacity needs, and implementation challenges. An optional open-ended follow-up item allowed respondents to provide their County/Region name if they wished. The instrument was designed for exploratory use within a specialised leadership population and was not intended as a validated measurement scale.

Ethical approval for the study was obtained from the University of the West Indies Campus Research Ethics Committee (CREC-SA.3717/02/2026) and the Ministry of Health, Trinidad and Tobago. Participation was voluntary, and informed consent was obtained electronically prior to survey completion. Responses were collected anonymously and analysed in aggregate to minimise the risk of identification given the small population size.

### 2.5 Data analysis

Closed-ended survey responses were analysed descriptively using frequencies and proportions. Given the small sample size, results were reported using counts (n) and percentages, and no inferential statistical analysis was conducted. Usefulness and importance items were rated on 1–5 scales, with higher scores indicating greater perceived usefulness or importance. Multiple-response items do not sum to 100%.

Open-ended responses were analysed using inductive thematic categorisation. Responses were reviewed to identify recurring patterns and key themes related to public health education, leadership decision-making, system preparedness, capacity-building needs, governance considerations, and implementation barriers. Due to the brief nature of responses, analysis focused on identifying common insights rather than conducting a formal qualitative thematic analysis.

Findings were interpreted in relation to the study objectives to provide an integrated understanding of leadership perspectives on the role of AI-enabled early warning systems in public health education, preparedness, and resilience.

## 3 Results

### 3.1 Participant characteristics

Six of the nine invited County Medical Officers of Health (CMOHs) completed the survey, representing a response rate of 66.7%. Participant characteristics, familiarity with artificial intelligence-enabled early warning systems (AI-EWS), and readiness for adoption are summarised in Table 1.

**Table 1:** Participant characteristics, familiarity, and readiness for AI-EWS (*n* = 6).

Variable	Category	*n* (%)
Years in leadership role	0–2 years	1 (16.7%)
	6–10 years	2 (33.3%)
	11+ years	3 (50.0%)
Familiarity with AI-EWS	Not familiar (1)	1 (16.7%)
	Slightly familiar (2)	2 (33.3%)
	Moderately familiar (3)	3 (50.0%)
Preparedness to adopt AI tools	Low (1, 2)	2 (33.3%)
	Moderate (3)	2 (33.3%)
	High (5)	2 (33.3%)
Estimated adoption timeframe	<1 year	1 (16.7%)
	1–2 years	3 (50.0%)
	3–5 years	2 (33.3%)

Three respondents (3/6) reported more than 11 years of experience in their current leadership role, two (2/6) reported 6–10 years, and one (1/6) reported less than 2 years. Familiarity with AI-EWS was generally low to moderate, with three respondents (3/6) reporting moderate familiarity, two (2/6) reporting slight familiarity, and one (1/6) reporting no familiarity.

Perceived readiness to adopt AI or predictive analytics tools varied across respondents, with two (2/6) reporting low readiness, two (2/6) reporting moderate readiness, and two (2/6) indicating higher readiness levels. Regarding adoption timelines, three respondents (3/6) indicated that AI-EWS could be adopted within 1–2 years if funding were available, two (2/6) indicated a timeframe of 3–5 years, and one (1/6) indicated adoption could occur within less than 1 year.

### 3.2 Current monitoring practices

Respondents reported using a combination of formal and informal mechanisms to monitor public health threats. Four respondents (4/6) identified community-based reporting, while three (3/6) referenced informal communication tools such as WhatsApp, email, or social media. One respondent (1/6) reported relying solely on established surveillance systems, and one (1/6) indicated no additional tools beyond routine systems.

### 3.3 Priority public health threats and system usefulness

All respondents (6/6) identified infectious diseases and flooding as priority public health threats for AI-EWS application. Three respondents (3/6) identified heatwaves, and one respondent (1/6) identified drought. Perceived usefulness of system features varied, with most respondents rating key features as useful or very useful. Short-term and long-term predictions were rated as useful or very useful by four respondents each (4/6). Automated alerts were rated as very useful by three respondents (3/6) and useful by two (2/6). Visual dashboards and integration with emergency services were rated as useful or very useful by five respondents each (5/6), while risk-level mapping was rated as very useful by three respondents (3/6) and useful by two (2/6).

A summary of key perceived applications and system features is presented in Table 2.

**Table 2:** Summary of perceived applications, system priorities, and barriers for AI-EWS.

Domain	Indicator	Result
Priority threats	Infectious diseases	6 (100%)
	Flooding	6 (100%)
	Heatwaves	3 (50%)
	Drought	1 (16.7%)
Usefulness of features	Short-term prediction useful/very useful	4 (66.7%)
	Long-term prediction useful/very useful	4 (66.7%)
	Automated alerts very useful	3 (50%)
	Dashboards useful/very useful	5 (83.3%)
	Risk mapping very useful	3 (50%)
	Integration useful/very useful	5 (83.3%)
Design priorities	Coordination very important	5 (83.3%)
	Risk thresholds very important	4 (66.7%)
	Timeliness important/very important	4 (66.7%)
	Data privacy important/very important	5 (83.3%)
Equity and governance	Equity prioritised	6 (100%)
	Equity in alert and response	5 (83.3%)
	Human oversight important	5 (83.3%)
	Multi-level alerts preferred	4 (66.7%)
Barriers	Budget constraints	5 (83.3%)
	Lack of technical skills	3 (50%)
	Data access limitations	3 (50%)
	Limited staff time	2 (33.3%)
	Governance/legal challenges	2 (33.3%)
	Lack of trust in AI	2 (33.3%)

### 3.4 Priority features for system design

Respondents identified several features as important or very important for future AI-EWS design. Coordination across health and emergency sectors was rated as very important by five respondents (5/6), while actionable risk thresholds were rated as very important by four respondents (4/6). Timeliness of alerts, user-friendly interfaces, and data privacy safeguards were each rated as important or very important by at least four respondents (4/6).

In ranking design principles, accessibility and integration with local context were most frequently prioritised. Additional considerations included affordability and usability within resource-constrained settings.

### 3.5 Equity, human oversight, and geographic scale

All respondents (6/6) agreed or strongly agreed that AI-EWS should prioritise equitable access, including reaching underserved and low-connectivity populations. Five respondents (5/6) indicated that equity should be addressed in both the alert and response phases. Human oversight was considered important, with five respondents (5/6) rating expert review of AI-generated alerts as important or very important. Regarding geographic scale, four respondents (4/6) preferred a multi-level system with drill-down capability, while two respondents (2/6) preferred regional or country-wide alerts.

### 3.6 Institutional readiness, training needs, and partnerships

Perceived readiness for AI adoption was mixed, reflecting variation in infrastructure, skills, and organisational capacity (Table 1). Respondents identified several areas requiring support, including training in AI tools (3/6), improved information technology infrastructure (3/6), increased staffing (2/6), and policy development or regulatory guidance (2/6).

All respondents (6/6) identified Regional Health Authorities as essential partners for implementation. Other frequently identified partners included municipal or local government (5/6), CARPHA and PAHO (4/6), academic institutions (4/6), NGOs and civil society groups (4/6), and international organisations (3/6).

### 3.7 Barriers to implementation

Key barriers to implementation are summarised in Table 2. The most frequently reported barrier was budget constraints, identified by five respondents (5/6). Additional barriers included lack of technical skills (3/6), inadequate data access (3/6), limited staff time (2/6), governance or legal challenges (2/6), and lack of trust in AI systems (2/6).

Open-ended responses emphasised the importance of infrastructure, training, and stakeholder engagement. One respondent noted that implementation would be constrained without adequate equipment and internet access, while another highlighted the need for capacity-building and organisational support to ensure sustainable adoption.

## 4 Discussion

### 4.1 Main findings

This study provides early empirical insight into County Medical Officers of Health (CMOHs) perspectives on artificial intelligence-enabled early warning systems (AI-EWS) in a Small Island Developing State context. The findings indicate that CMOHs recognise the potential value of AI-EWS not only as tools for disease prediction and surveillance, but also as mechanisms to support public health decision-making, coordination, and professional learning.

Despite relatively low to moderate familiarity with AI-EWS, respondents consistently identified key public health applications, including infectious disease surveillance and flood-related health risks. At the same time, findings highlight important gaps in institutional readiness, particularly in relation to infrastructure, technical capacity, and governance frameworks. These results suggest that while interest in AI-enabled systems exists, implementation will depend on targeted investments in capacity building and system-level preparedness.

### 4.2 AI-EWS as tools for decision-making and public health education

A key contribution of this study is the identification of AI-EWS as not solely predictive technologies, but as tools that can support public health education, leadership decision-making, and system-level learning. Respondents highlighted the value of features such as automated alerts, dashboards, and risk mapping, which can facilitate interpretation of complex data and support timely decision-making.

This aligns with emerging literature suggesting that AI can enhance public health practice by enabling real-time data analysis, improving situational awareness, and supporting evidence-based decision-making (9, 12). Recent Frontiers research also highlights the potential role of AI in public health education, including its capacity to support learning, communication, and the interpretation of complex public health information (14). However, the findings extend this perspective by emphasising the role of these systems in professional learning and capacity building. In this context, AI-EWS may function as practical tools for training, scenario-based planning, and strengthening institutional knowledge within public health systems, particularly in resource-constrained settings.

The findings also align with emerging work on AI-supported public health decision-making, which suggests that AI may assist leaders and public health actors by improving access to timely evidence during routine and crisis decision contexts (13). However, the small number of applied studies in this area reinforces the need for context-specific evidence from public health leaders, particularly in SIDS and other resource-constrained settings.

### 4.3 Readiness and capacity constraints in SIDS contexts

The findings highlight uneven readiness for AI adoption among CMOHs, reflecting broader structural challenges within SIDS health systems. Key constraints identified include limited technical skills, insufficient data infrastructure, and resource limitations, particularly in relation to equipment and connectivity.

These findings are consistent with existing literature highlighting the importance of digital infrastructure, workforce capacity, and governance frameworks in enabling the effective use of AI in public health (10, 12). They also align with global guidance on climate-resilient health systems, which identifies governance, health workforce capacity, climate-informed surveillance, early warning systems, infrastructure, and financing as important components of climate-resilient health system strengthening (17). In the context of SIDS, these challenges may be amplified due to smaller human resource bases, financial constraints, and existing system pressures related to climate and health risks. This is consistent with emerging frameworks on artificial intelligence readiness in public health, which emphasise governance, infrastructure, data systems, and workforce capacity as foundational requirements for implementation (16).

Most importantly, the identification of training needs and infrastructure gaps suggests that readiness for AI-EWS is not solely a technological issue, but a broader system-level challenge requiring coordinated efforts across policy, training, and organisational domains. For Caribbean health systems, this reinforces the need to connect AI-enabled innovation with wider climate-health preparedness, surveillance strengthening, and institutional capacity-building efforts (3, 5, 17, 18).

Several technical challenges are particularly relevant to AI-EWS implementation in SIDS contexts. These include fragmented data systems, incomplete or delayed surveillance data, limited interoperability between health, meteorological, environmental, and emergency management systems, uneven internet connectivity, and insufficient local capacity for model development, validation, maintenance, and interpretation. In small populations, additional challenges may arise from sparse datasets, limited historical event data, and difficulties validating predictive models across diverse geographic or hazard contexts. These issues can affect the reliability, timeliness, and usability of AI-generated alerts. Addressing these challenges will require staged implementation, investment in data governance and interoperability, strengthening of routine surveillance and climate-health data systems, local validation of models before operational use, and sustained training for public health teams who will interpret and act on AI-EWS outputs.

### 4.4 Equity, governance, and responsible AI implementation

Equity emerged as a central concern, with all respondents emphasising the importance of ensuring that AI-EWS reach underserved and low-connectivity populations. This finding reinforces the need to design AI systems that are inclusive and context-sensitive, particularly in settings where digital divides may exacerbate existing health inequalities.

This is consistent with recent guidance on integrating health equity into AI for public health, which emphasises that equity must be considered across the AI lifecycle, including data collection, model development, implementation, evaluation, and governance (15). Respondents also highlighted the importance of human oversight, reflecting concerns related to trust, accountability, and the reliability of AI-generated outputs. These perspectives align with broader discussions on ethical AI in public health, which emphasise the need for transparency, governance frameworks, and safeguards to ensure responsible use.

Taken together, these findings underscore that the successful implementation of AI-EWS will depend not only on technical performance, but also on the integration of ethical principles, governance mechanisms, and equity considerations into system design and deployment. Several ethical dilemmas may arise if AI-EWS are implemented without appropriate safeguards. These include privacy risks related to the integration of health, environmental, mobility, or community-level data; algorithmic bias if training data underrepresent rural, low-connectivity, or underserved populations; false reassurance if model outputs are treated as definitive; alert fatigue if warnings are too frequent or poorly targeted; and accountability concerns if it is unclear who is responsible for acting on AI-generated alerts.

In SIDS contexts, these concerns are especially important because uneven connectivity, resource constraints, and differences in local response capacity may affect who benefits from early warnings and who remains underserved. Mitigation strategies should therefore include privacy-preserving data practices, transparent governance arrangements, human oversight of alerts, regular model evaluation, clear escalation protocols, inclusive communication strategies, and mechanisms for community and stakeholder feedback.

### 4.5 Implications for policy and practice

The findings have several implications for public health policy and practice in SIDS contexts. First, there is a need for targeted investment in training and capacity building to support the adoption of AI-enabled tools among public health professionals. Second, strengthening digital infrastructure, including access to reliable internet, data systems, and equipment, will be critical for enabling effective implementation.

Third, the importance of partnerships identified by respondents suggests that multi-sectoral collaboration, across health authorities, academic institutions, regional organisations, and international agencies, will be essential for scaling AI-EWS. This aligns with disaster risk reduction and climate-resilient health system guidance, which emphasise coordinated governance, preparedness, early warning capacity, and cross-sectoral collaboration (7, 17). Finally, the emphasis on equity and governance highlights the need for policy frameworks that support responsible and inclusive AI adoption, ensuring that technological advancements translate into improved public health outcomes across all population groups.

For Trinidad and Tobago and similar SIDS settings, AI-EWS should therefore be introduced as part of a broader health system strengthening agenda rather than as a stand-alone technological intervention. A practical pathway would include beginning with priority use cases such as infectious disease surveillance and flood-related health risks, strengthening data-sharing arrangements across health, meteorological, environmental, and emergency management agencies, developing clear governance and accountability protocols, and piloting AI-EWS tools with human oversight before wider scale-up. Implementation should also include training for public health teams, investment in interoperable data systems, and communication strategies that ensure alerts are accessible to underserved and low-connectivity communities. These steps would help address the technical and ethical challenges identified in this study while supporting responsible, context-appropriate adoption.

### 4.6 Strengths and limitations

This study has several strengths. It provides an important empirical insight from a highly specialised leadership group directly involved in public health decision-making. By targeting all CMOHs within Trinidad and Tobago, the study captures perspectives from key actors responsible for translating surveillance data into policy and operational action.

However, the study also has limitations. The small sample size reflects the unique number of CMOHs in the country and restricts the generalisability of findings. In addition, data were self-reported and not independently verified, introducing the potential for information or reporting bias. Open-ended responses were brief, limiting the depth of qualitative analysis. Despite these limitations, the study provides valuable exploratory insight and establishes a foundation for future research, including larger multi-country studies and more in-depth qualitative investigations.

## 5 Conclusion

This study demonstrates that CMOHs in Trinidad and Tobago recognise the potential of AI-enabled early warning systems as tools for enhancing public health preparedness, decision-making, and professional learning. At the same time, successful implementation will require addressing gaps in infrastructure, data quality, interoperability, technical capacity, governance, and equity. As climate-related health risks intensify in SIDS contexts, AI-EWS present an opportunity to strengthen anticipatory public health systems.

To realise this potential, AI-EWS should be introduced through a phased, human-centred approach that begins with priority use cases, strengthens data-sharing and governance arrangements, validates models in local contexts, builds workforce capacity, and ensures that alert and response mechanisms reach underserved and low-connectivity populations. Such an approach would allow AI-EWS to support public health preparedness while reducing risks related to bias, privacy, overreliance, and inequitable implementation.

## Data Availability

The original contributions presented in the study are included in the article/Supplementary material, further inquiries can be directed to the corresponding author.

## References

[1] Intergovernmental Panel on Climate Change. Climate Change 2022: Impacts, Adaptation and Vulnerability. Cambridge: Cambridge University Press (2023).

[2] Pan American Health Organization. Climate Change and Health in the Caribbean: An Agenda for Action. Washington: Pan American Health Organization (2025).

[3] Rise N Oura C Drewry J. Climate change and health in the Caribbean: a review highlighting research gaps and priorities. J Clim Change Health. (2022) 8:100126. doi: 10.1016/j.joclim.2022.100126

[4] UK Government. Small Island Developing States: Vulnerability Note. London: GOV.UK (2025).

[5] Pan American Health Organization. Health and Climate Change: Country Profile 2025—Trinidad and Tobago. Washington: Pan American Health Organization (2026).

[6] United Nations Office for Disaster Risk Reduction. Early Warnings for All Initiative. Geneva: United Nations Office for Disaster Risk Reduction (2025).

[7] United Nations Office for Disaster Risk Reduction. Sendai Framework for Disaster Risk Reduction 2015–2030. Geneva: United Nations Office for Disaster Risk Reduction (2015).

[8] World Health Organization. Early Warning, Alert and Response System in Emergencies: Operational guide. Geneva: World Health Organization (2023).

[9] Villanueva-Miranda I Xiao G Xie Y. Artificial intelligence in early warning systems for infectious disease surveillance: a systematic review. Front Public Health. (2025) 13:1609615. doi: 10.3389/fpubh.2025.1609615, 40626156 PMC12230060

[10] Mendes VIS Mendes BMF Moura RP Lourenço IM Oliveira MFA Ng KL . Harnessing artificial intelligence for enhanced public health surveillance: a narrative review. Front Public Health. (2025) 13:1601151. doi: 10.3389/fpubh.2025.1601151, 40809756 PMC12343694

[11] Olawade DB Wada OJ David-Olawade AC Kunonga E Abaire O Ling J. Using artificial intelligence to improve public health: a narrative review. Front Public Health. (2023) 11:1196397. doi: 10.3389/fpubh.2023.1196397, 37954052 PMC10637620

[12] Panteli D Adib K Buttigieg S Goiana-da-Silva F Ladewig K Azzopardi-Muscat N . Artificial intelligence in public health: promises, challenges, and an agenda for policy makers and public health institutions. Lancet Public Health. (2025) 10:e428–32. doi: 10.1016/S2468-2667(25)00036-2, 40031938 PMC12040707

[13] Campbell EA Goodtree H Gillani S Oyefolu O Kelly A Rivers C . Impact, use, and implications of artificial intelligence in public health decision making by elected officials: a scoping review. Front Public Health. (2026) 14:1745684. doi: 10.3389/fpubh.2026.1745684, 41853148 PMC12992293

[14] Wang J Li J. Artificial intelligence empowering public health education: prospects and challenges. Front Public Health. (2024) 12:1389026. doi: 10.3389/fpubh.2024.1389026, 39022411 PMC11252473

[15] Ghanem S Moraleja M Gravesande D Rooney J. Integrating health equity in artificial intelligence for public health in Canada: a rapid narrative review. Front Public Health. (2025) 13:1524616. doi: 10.3389/fpubh.2025.1524616, 40171421 PMC11958991

[16] Pan American Health Organization, Inter-American Development Bank. Artificial Intelligence in Public Health: Readiness Assessment Toolkit. Washington: Pan American Health Organization (2024).

[17] World Health Organization. Operational Framework for Building Climate Resilient and Low Carbon Health Systems. Geneva: World Health Organization (2023).

[18] Drewry J Oura CAL. Strengthening climate resilient health systems in the Caribbean. J Clim Change Health. (2022) 6:100135. doi: 10.1016/j.joclim.2022.100135

