# Supplementary material for: Artificial intelligence-enabled early warning systems for public health preparedness: perspectives of senior public health leaders in a Small Island Developing State
Source: Front Public Health. 2026 Aug 13;14:1931303. doi: 10.3389/fpubh.2026.1931303 (PMC13518320; doi:10.3389/fpubh.2026.1931303)
Supplement: Supplementary file 1 [file Data_Sheet_1.pdf]

Supplementary Material 1
Survey Instrument

Survey on Leadership Perspectives on AI-Enabled Early Warning Systems (AI-EWS) in Public Health - Trinidad and Tobago

This document presents the survey instrument used in the study. It has been formatted as a supplementary material file for journal submission. No participant responses are included.

Study Information and Consent

Artificial Intelligence (AI) is rapidly reshaping how public health systems anticipate, monitor and respond to emerging threats. From climate-related disasters to disease outbreaks, AI-enabled Early Warning Systems (AI-EWS) offer a powerful tool for forecasting risks, supporting decision-making, and protecting communities, especially in vulnerable settings like Small Island Developing States (SIDS).

Purpose

This study seeks to explore leadership perspectives on the potential of AI-EWS to support public health preparedness and resilience across Trinidad and Tobago. Specifically, it investigates the level of awareness, perceived usefulness, design priorities, governance concerns, and readiness among public health decision-makers.

Survey Overview

You are invited to participate in a brief survey examining the role of AI-EWS in strengthening public health in Trinidad and Tobago. The survey includes a total of 20 questions, with a mix of multiple-choice, scaled questions and open-ended questions across several themes including system design, ethics, equity, training needs, and barriers to implementation. It will take approximately 10 to 12 minutes to complete.

Note: The survey has been approved by The UWI Campus Ethics Committee (CREC-SA.3717/02/2026) and the Ministry of Health, with approval from the Office of the Chief Medical Officer (22-04-2026).

Participation

Your participation is entirely voluntary. You may decline to answer any question or exit the survey at any time without penalty.

Benefits

There are no direct personal benefits to you. However, your insights will contribute to better understanding how future health alert systems can be more effective, inclusive, and responsive to the needs of Trinidad and Tobago's public health leadership.

Risks

There are no known risks beyond minor time inconvenience. No personal or sensitive data is being collected.

Confidentiality

Your responses are anonymous and reported only in aggregate form. No identifying information will be collected or published. Survey data will be stored on a password-protected system, accessible only to the research team.

Contact

If you have any questions about this study or your rights as a participant, you may contact:

Principal Investigator: Dr. Letetia Addison - letetia.addison@sta.uwi.edu

UWI Campus Research Ethics Committee: STA-researchethics@sta.uwi.edu

Consent. By checking the box below, you acknowledge that you have read and understood the above information and that: *

- Your participation in this survey is voluntary. You may withdraw your consent and discontinue participation in the survey completion process at any time. Your refusal to participate will not in any way adversely impact you.

- You have given consent to be a participant of this research and respond to the survey/questionnaire(s) as truly as possible.

☐ I agree to participate in this survey.

Survey Questions

The survey comprises 20 questions, with multiple choice, scaled questions and open-ended questions across several themes in mini Sections A to H.

Section A - Leadership Background

This section asks about your leadership experience and the tools currently used to monitor public health threats.

1. Years spent in current leadership role (e.g., CMOH or equivalent). *

Mark only one option.

○ 0-2 years

○ 3-5 years

○ 6-10 years

○ 11+ years

2. Besides established surveillance systems, are there additional tools or systems your team uses to anticipate or monitor public health threats? (e.g., mobile outbreak tracking apps, informal WhatsApp alerts, surveillance dashboards, weather or environmental alerts, community-based reporting, etc.) *

____
____

____
____

____
____

____
____

Section B - Knowledge, Awareness and Use

This section explores your familiarity with AI-enabled early warning systems and their potential applications.

Key definitions used in the survey:

- Early Warning System (EWS): Uses data to forecast or detect public health threats and alert decision-makers.
- AI-enabled EWS (AI-EWS): Enhances EWS capabilities using artificial intelligence (e.g., forecasting outbreaks by integrating hospital, weather, or mobility data).

The following question uses the scale points shown to rate familiarity.

Rating	Meaning in this context
1 - Not at all familiar	You have never heard of AI-EWS and have no knowledge of what they are or how they work.
2 - Somewhat / Slightly familiar	You have heard of AI-EWS in passing or have a very basic idea, but little understanding of their functions or applications.
3 - Moderately familiar (Neutral)	You know what AI-EWS are and may have read or heard about them, but you have not worked directly with one or applied them in practice.
4 - Familiar	You have a good understanding of AI-EWS concepts and may have some experience using or evaluating them in a professional context.
5 - Very familiar	You have strong knowledge and direct experience with AI-EWS, including practical use, implementation, or research in public health.

3. How familiar are you with AI-enabled Early Warning Systems for public health events? (1 = Not at all familiar; 5 = Very familiar. Refer to previous table.) *

Mark only one option.

○ 1 - Not at all familiar

○ 2 - Slightly familiar

○ 3 - Moderately familiar

○ 4 - Familiar

○ 5 - Very familiar

4. Which types of public health threats would benefit most from AI-EWS in your county? (Select all that apply) *

Tick all that apply.

☐ Infectious diseases (e.g., dengue, leptospirosis, malaria)

☐ Flooding

☐ Heatwaves

☐ Drought

☐ Other: ______________________________

The following question uses the scale points shown to rate usefulness.

Rating	Meaning in this context
1 - Not useful	The feature does not contribute to decision-making and would not be applied in your work.
2 - Slightly useful	The feature adds minor value; it may help occasionally but is not central to decisions.
3 - Moderately useful (Neutral)	The feature is somewhat helpful, but not clearly essential. It may be relevant in certain contexts but not consistently relied upon.
4 - Useful	The feature provides clear value and would regularly inform or improve decision-making.
5 - Very useful	The feature is critical; it would strongly enhance decision-making and should be prioritized in AI-EWS design.

5. How useful would each of the following AI-EWS features be for decision-making in public health? (1 = Not useful; 5 = Very useful. Refer to previous table.) *

Mark only one option per row.

Feature	1 - Not useful	2 - Slightly useful	3 - Moderately useful	4 - Useful	5 - Very useful
Short-term prediction (e.g., days/weeks)	☐	☐	☐	☐	☐
Long-term prediction (e.g., months)	☐	☐	☐	☐	☐
Automated alerts	☐	☐	☐	☐	☐
Visual dashboards	☐	☐	☐	☐	☐
Risk-level mapping	☐	☐	☐	☐	☐
Integration with emergency services	☐	☐	☐	☐	☐

Section C - Design Priorities

This section explores your views on which system features should be prioritised in future AI-EWS design.

The following question uses the scale points shown to rate importance of system features.

Rating	Meaning in this context
1 - Not important	This feature is not needed at all in an AI-EWS.
2 - Somewhat important	The feature adds minor value but is not central to system design.
3 - Moderately important (Neutral)	The feature is useful in some cases, but not a top priority compared to others.
4 - Important	The feature should be included and will significantly improve system effectiveness.
5 - Very important	The feature is essential and must be prioritized in AI-EWS design.

6. How important are the following features in a future AI-EWS? (1 = Not important; 5 = Very important. Refer to previous table.) *

Mark only one option per row.

Feature	1 - Not important	2 - Slightly important	3 - Moderately important	4 - Important	5 - Very important
Timeliness of alerts (in terms of speed and responsiveness).	☐	☐	☐	☐	☐
Simple, user-friendly visual interface (in terms of usability and adoption potential).	☐	☐	☐	☐	☐
Actionable risk thresholds (in terms of decision-making utility).	☐	☐	☐	☐	☐
Coordination across health and emergency sectors (in terms	☐	☐	☐	☐	☐

of system-level integration).					
Data privacy and security safeguards (in terms of safeguarding sensitive patient health data).	☐	☐	☐	☐	☐

7. Which design principles should be prioritised for AI-EWS? (Please rank the following from 1 (your top priority) to 5 (your lowest priority)). *

Select one number per row.

Note: You can only select one number per column in order of priority, e.g., Accessibility - 1, Mobile-first design - 4, Multilingual Interface - 5, Equity for marginalised groups - 2, Integration with local context - 3.

**Design principle**	**1**	**2**	**3**	**4**	**5**
Accessibility	☐	☐	☐	☐	☐
Mobile-first design	☐	☐	☐	☐	☐
Multilingual interface	☐	☐	☐	☐	☐
Equity for marginalised groups	☐	☐	☐	☐	☐
Integration with local context	☐	☐	☐	☐	☐

8. Are there any other design principles that you believe should be prioritised for AI-EWS? (If there are please state, otherwise indicate N/A). *

____
____
____
____

Section D - Governance and Ethics

This section examines perspectives on oversight, equity, and ethical considerations in AI-EWS.

9. How should an AI-EWS balance the trade-off between speed and accuracy of alerts? *

Mark only one option.

○ Prioritize speed over perfect accuracy

○ Prioritise high accuracy even if alerts are slower

○ Balance both speed and accuracy equally

○ Do not know/Not sure

10. To what extent do you agree: "AI-EWS must prioritise equity and ensure reach to rural, low-connectivity, and underserved populations." (1 = Strongly Disagree; 5 = Strongly agree) *

Mark only one option.

- ○ Strongly Disagree
- ○ Disagree
- ○ Neutral
- ○ Agree
- ○ Strongly Agree

11. In your view, should equity be addressed primarily in the alert phase, the response phase, or both? *

Mark only one option.

- ○ Alert phase
- ○ Response phase
- ○ Both alert phase and response phase
- ○ Do not know/Not sure

The following question uses the scale points shown to rate importance.

Rating	Meaning in this context
1 - Not important	AI alerts can be trusted without human review.
2 - Slightly important	Human oversight is occasionally useful but not critical.
3 - Moderately important (Neutral)	Human oversight adds value but alerts could still be used without constant review.
4 - Important	Expert review is needed to ensure alerts are reliable and actionable.
5 - Very important	Human oversight is essential; AI alerts should always be validated before use.

12. How important is human oversight (expert review) of AI-generated alerts for quality and accuracy? (1 = Not important; 5 = Very important. Refer to table) *

Mark only one option.

- ○ 1 - Not important
- ○ 2 - Slightly important
- ○ 3 - Moderately important
- ○ 4 - Important
- ○ 5 - Very important

13. At what geographic scale should an AI-EWS ideally generate alerts? (Select the one you believe is most appropriate.) *

Mark only one option.

Note: Multi-level means that the system provides alerts at national, regional, and local levels, with the ability to zoom in or out depending on the situation).

- ○ Small area (e.g., neighborhood, village)
- ○ Regional or country-wide
- ○ National
- ○ Multi-level with drill-down capability
- ○ Do not know/Not sure

Section E - Capacity and Training

This section looks at your team's readiness and support needs for adopting AI-EWS tools.

The following question uses the scale points shown to rate preparedness.

Rating	Meaning in this context
1 - Not prepared	No readiness; little awareness, skills, or infrastructure.
2 - Slightly prepared	Minimal exposure or partial resources; limited capacity.
3 - Moderately prepared (Neutral)	Some skills or infrastructure exist, but significant gaps remain. Adoption possible with support.
4 - Prepared	Team is generally ready; most skills and infrastructure are in place, only minor adjustments needed.
5 - Very prepared	Team is fully ready; strong technical capacity, infrastructure, and confidence to adopt AI tools.

14. How prepared is your team to adopt AI or predictive analytics tools for public health? (1 = Not prepared; 5 = Very prepared. Refer to previous table.) *

Mark only one option.

○ 1 - Not prepared

○ 2 - Slightly prepared

○ 3 - Moderately prepared

○ 4 - Prepared

○ 5 - Very prepared

15. What training, resources, or support would best help your team adopt AI-EWS tools? *

____
____

____
____

____
____

____
____

Section F - Adoption and Partnerships

This section focuses on organisational changes and partnerships that could enable AI-EWS implementation.

16. What changes would help your county prepare for adopting AI-EWS tools (e.g., in staffing, policy, or systems)? *

____
____

____
____

____
____

____
____

17. Which local, regional, or international partners would be essential to successful AI-EWS implementation? (Select all that apply) *

Tick all that apply.

☐ Regional Health Authorities (RHAs)

☐ County Medical Officers of Health (CMOHs)

☐ Municipal/local government

☐ NGOs / civil society groups

☐ CARPHA

☐ PAHO

☐ CDEMA

☐ WHO

☐ UN entities (UNICEF / UNDP / UNESCO etc)

☐ CDC (USA)

☐ Academic/research institutions

☐ Donor agencies (World Bank, IDB)

☐ Other: ______________________________

18. If funding were available, in what timeframe could your organisation realistically adopt AI-EWS tools? *

Mark only one option.

○ Less than 1 year

○ 1-2 years

○ 3-5 years

○ More than 5 years

Section G - Barriers and Enablers

This section identifies challenges and key actions that could influence successful adoption of AI-EWS.

19. What are the biggest barriers to implementing AI-EWS in public health? (Select your top three (3) selections) *

Tick all that apply.

☐ Budget constraints

☐ Limited staff time

☐ Lack of technical skills

☐ Inadequate data access

☐ Legal or governance challenges

☐ Lack of trust in AI systems

☐ Public communication issues

☐ Other: ______________________________

20. In your opinion, what one action would most improve the success of AI-EWS in Trinidad & Tobago? *

____

____

Section H - Follow-Up (Optional)

This section allows you to provide additional information about your county or region if you wish.

Optional. Would you be willing to share your County/Region name? (If yes, please state below, otherwise proceed to the end of the survey).
